# Effect of Sterols Isolated from *Myrtillocactus geometrizans* on Growth Inhibition of Colon and Breast Cancer Cells

**DOI:** 10.1155/2015/589350

**Published:** 2015-05-31

**Authors:** Mario Augusto Bolaños-Carrillo, Jose Luis Ventura-Gallegos, Arturo David Saldivar-Jiménez, Alejandro Zentella-Dehesa, Mariano Martínez-Vázquez

**Affiliations:** ^1^Instituto de Química, Universidad Nacional Autónoma de México, 04510 México, DF, Mexico; ^2^Instituto de Investigaciones Biomédicas, Universidad Nacional Autónoma de México, 04510 México, DF, Mexico; ^3^Departamento de Bioquímica, Instituto Nacional de Ciencias Médicas y Nutrición “Salvador Zubirán”, 14080 México, DF, Mexico

## Abstract

*Objective*. To explore the effect of peniocerol and macdougallin on HCT-15 and MCF-7 cells proliferation, cell cycle, apoptosis, and PARP cleavage. *Methods*. HCT-15 and MCF-7 cells were treated with various concentrations of peniocerol and macdougallin (10–80 *μ*M) during 24 or 48 h. Crystal Violet Assay was used to evaluate the inhibition effect. Cell cycle regulation was examined by a propidium iodide method. Cell apoptosis was detected through both Annexin–V FLUOS/PI double-labeled cytometry assays and Western blot was applied to assess PARP cleavage. *Results*. Peniocerol and macdougallin induced growth inhibition and apoptosis *in vitro* in a time- and dose-dependent manner. Moreover, peniocerol and macdougallin induced arrest of cell cycle-dependent manner and increased the proportion of cells in G_0_/G_1_ phase. PARP cleavage in HCT-15 and MCF-7 cells was induced by treatment with peniocerol and macdougallin after 36 hours. *Conclusions*. Our results showed that the mechanism of cytotoxicity displayed by peniocerol and macdougallin is related to cell cycle arrest and apoptosis in both cell lines. This is a significant observation because it helps to understand the way some oxysterols isolated from *Myrtillocactus geometrizans* develop their biological activities against cancer cells.

## 1. Introduction

Cholesterol (**1**), the main sterol in animals, has been associated with atherosclerosis, while the phytosterols, sterols synthesized by plants, have been proven to reduce serum cholesterol levels, decreasing cardiovascular risk [1]. The most abundant phytosterols are *β*-sitosterol (**2**) and campesterol; stigmasterol and brassicasterol are less common. Cholesterol and phytosterols both have a steroid nucleus and a hydroxyl at C-3. However, due to the presence of a double bond between C-5 and C-6, sterols can undergo oxidative processes [2]. In contrast, plant stanols do not have this double bond and are unlikely to oxidize. Nevertheless, the only structural difference between cholesterol and phytosterols resides in the side chain located in C-17. Compared to cholesterol, phytosterols have different substitutions in C-24 [2]. These structural differences could account for their quite different biological activities. The oxidation of the C5–C6 double bond, or of those in other positions in the steroid ring or side chain of sterols, results in the formation of oxysterols or sterol oxidation products (SOPs). In the case of cholesterol oxidation, the resulting products are usually named COPs (cholesterol oxidation products) and, when phytosterols are oxidized, the resulting products are named POPs (phytosterol oxidation products). COPs are now thought to be potentially involved in the initiation and progression of major chronic diseases including atherosclerosis, neurodegenerative processes, diabetes, kidney failure, and ethanol intoxication [3, 4]. Even though POPs have been associated with atherosclerosis diseases and cytotoxic effects, this has only been found at concentrations [5, 6]. Due to the ability of phytosterol compounds to reduce plasma serum cholesterol levels in humans, their addition to foods has increased significantly over the past decade [7]. However, there is some concern as these compounds showed cytotoxic activity towards normal cells.

On the other hand, there are numerous references about the phytosterols cytotoxicity activities against several human cancer cells [8, 9]. For example, it has been reported that** 2** inhibited the MDA-MB-231 cell growth by 70% compared with control and induced cell cycle arrest at the G2/M phase. In contrast, cholesterol treatment to the same cells increased cellular migration [10].

In another study, the dietary effect of phytosterols versus cholesterol on the growth and metastasis of the MCF-7 human cancer cells in SCID mice was evaluated. The results showed that animals fed with phytosterols diet had tumors 40–43% smaller than those fed with the cholesterol diet [11]. However, in a recent study, it was shown that the intake of phytosterols accelerated intestinal tumorigenesis in Apc^Min^ mice; this effect was stronger in female mice [12].

Peniocerol (**3**) is a sterol isolated from* Myrtillocactus geometrizans.* Unlike the structure of** 2**, peniocerol has the same lateral chain at C-17 as cholesterol; however, it has an alcohol group at C-6 and a double bond in C-8/C-9, while cholesterol has a double bond at C-5/C-6. Beyond these differences, both structures are identical. The absence of a double bond at C-5/C-6 prevents the oxidation of** 3** as common in cholesterol, although the presence of an alcohol group at C-6 would indicate a degree of oxidation. Despite its structural similarity to cholesterol, we have shown the anti-inflammatory and antiproliferative properties of** 3**; such activities are more common to phytosterols than to cholesterol [13].

Furthermore, as a result of a previous study about the activity of** 3** on mitochondria, we postulated that the cytotoxic activity of this sterol could be attributable to the oxidation of critical thiols located on adenine nucleotide translocase, the protein mainly involved in permeability transition pores (PTP). This event in the presence of Ca^2+^ induces the mitochondrial permeability transition (MPT) with the release of the proapoptotic factors cytochrome c and apoptosis-inducing factor (AIF). These observations evidence that peniocerol may trigger both the caspase-dependent and caspase-independent apoptotic pathways [14].

In order to provide data to support the proposal on apoptosis induced by peniocerol (**3**), we decided to evaluate the cytotoxic activity and determine the cell death type as well as the probable disruption of the cell cycle by** 3** in the HCT-15 and MCF-7 human cancer lines.

The sterol macdougallin (**4**) is also isolated from* M. geometrizans*. It is significant that the only structural difference between** 3** and** 4** is the presence of *α*-methyl group at C-14 in the latter. Therefore, in order to find if this small structural difference could account for its biological activities, we decided to also evaluate** 4**.

## 2. Materials and Methods

### 2.1. Chemicals

All antibodies were purchased from Santa Cruz Biotechnology (Santa Cruz, CA). Crystal Violet reagent, RPMI-1640, fetal bovine serum (FBS), Annexin V-FLUOS Staining kit, Propidium Iodide (PI), and RNase A were purchased from Roche (Manheim, Germany).

### 2.2. Extraction and Isolation

Peniocerol, (3*β*, 6*α*-diol-cholest-8-ene) and Macdougallin (14*α*-methyl-3*β*, 6*α*-diol-cholest-8-ene) were isolated from* Myrtillocactus geometrizans* and purified as previously described [13]. Copies of the original spectra are obtainable from the author.

### 2.3. Cell Lines and Cell Culture

Colon adenocarcinoma (HCT-15) and breast adenocarcinoma (MCF-7) were obtained from ATCC. Medium contained 10% fetal bovine serum, penicillin 100 IU/mL, and streptomycin 100 g/mL, RPMI, at 37°C, 5% CO_2_, and water saturated humidity condition. Every 1 or 2 days, for a fluid passage, the logarithm growth period of cell activity of more than 95% cells was used in this study.

### 2.4. Cytotoxic Assay (Crystal Violet Assay)

The cytotoxicity assays were performed by seeding cells in 48-well plates at density of 2 × 10^4^ cells/cm^2^ in RPMI phenol red supplemented with 10% FBS, 2 mM L-Glutamine, 100 IU mL^−1^ Penicillin G, 100 *μ*g mL^−1^ streptomycin sulfate, and 0.25 of *μ*g mL^−1^ amphotericin B. The cell line cultures were maintained in a 5% CO_2_ and 95% humidity atmosphere at 37°C. The next day, a defined concentration of peniocerol or macdougallin was added. Cell viability was evaluated 24 and 48 h after treatment by Crystal Violet Assay. Test substances were dissolved in DMSO to create a stock solution and Doxorubicin was used as a positive control. DMSO was added to control wells at the highest concentration used and no effect in cell growth was observed (<1%). After incubation with test compounds was over, adherent cell cultures were fixed by adding 100 *μ*L of Glutaraldehyde 1.1% (wt/vol) and incubated at room temperature for 15 minutes. The supernatant was discarded and the places were washed with water and left to dry in the air. The fixed cells were stained with 100 *μ*L of Crystal Violet dye and the protein-bonded dye was solubilized with 200 *μ*L of 10% acetic acid (wt/vol). The values of optical density were read on a microplate reader (Elx808; BioTek Instruments, Inc., Winooski, VT, USA) with a test wavelength of 595 nm. A dose-response curve was plotted for each compound, and the IC_50_ was estimated from nonlinear regression using STATA 11 software (version 11.1; Stata Corporation, College Station, TX, USA).

### 2.5. Cell Cycle Assay

HCT-15 and MCF-7 cells were cultured to a density of 1 × 10^6^ cells in 100 × 20 mm culture petri dishes. After a 24 h period, cells were cultured in the presence of peniocerol and macdougallin at 40 *μ*M and 60 *μ*M concentrations during 24 hours. Timidine and Nocodazole were used as positive controls. After these treatment cells were harvested and centrifuged for five minutes at 1500 rpm, the pellet was resuspended in PBS (pH = 7.4) and centrifuged again for five minutes at 1500 rpm. Cells were fixed with 70% of methanol at 4°C for at least 24 h. Methanol was eliminated by centrifugation and the pellet was washed with PBS. DNA was labeled with 5 *μ*g/mL^−1^ Propidium Iodide (PI) solution. Cell cycle analysis was made using a FACScan cytometer (BD Biosciences, San Jose, CA) and CELLQuest software (BD Biosciences). The cell cycle profile was obtained by analyzing 10,000 events using the FlowJo software version 7.2.5 (Tree Star Inc, Ashland, OR, USA).

### 2.6. Annexin V-FITC Apoptosis Assay

Annexin V-FLUOS (Roche) and Propidium Iodide were used to measure apoptosis according to manufacturer's protocol. This assay measures the apoptotic cells by binding phosphatidylserine exposed on the cytoplasmic surface of the cell membrane of apoptotic cells. On the other hand, Propidium Iodide (PI) is a membrane impermeant dye that is generally excluded from viable cells. It binds to double stranded DNA by intercalating between base pairs of death cells. PI is excited at 488 nm and is emitted at a maximum wavelength of 617 nm. Briefly, 2 × 10^6^ HCT-15 cells were plated to each well plate. After 24 h, cells were treated with either DMSO alone at the highest concentration used as control, 40 *μ*M or 60 *μ*M of peniocerol, or 40 *μ*M or 60 *μ*M of macdougallin, respectively. At the end of the treatment, adherent and nonadherent cells were harvested and washed twice with PBS and then resuspended with 0.1 mL^−1^ of Annexin-binding buffer. Cells were incubated in the dark during 15 minutes at room temperature. After incubation, the samples were analyzed with FACScan flow cytometry (BD Biosciences, San Jose, CA) using CELLQuest Software (BD Biosciences). The percentage of apoptotic cells in the cell samples was obtained by analyzing 10,000 events using the FlowJo software version 7.2.5 (Tree Star Inc, Ashland, OR, USA).

### 2.7. Western Blot Analysis

Cells were treated with 40 *μ*M of peniocerol or macdougallin, respectively, for 12, 24, and 36 h and lysed in buffer containing 50 mM HEPES (pH 7.4), 1 mM EDTA, 250 mM NaCl, 1% Nonidet P-40, 10 mM NaF, 1 mM sodium vanadate, and protease cocktail inhibitor (complete, EDTA-free, Roche Diagnostics, Indianapolis, IN). Thirty micrograms of protein was run in SDS-PAGE and transferred to Immobilon-P PVDF membranes (Millipore Corp., Bedford, MA). The membranes were incubated with 5% dehydrated skim milk to block nonspecific protein bindings and then incubated with primary antibodies at room temperature overnight. The primary anti-human PARP-1, *α*-tubulin, and *β*-actin antibodies were used. Anti-rabbit antibodies were used as secondary antibodies. The blots were revealed using SuperSignal West Pico Chemiluminescent Substrate (Thermo Scientific, Rockford, IL). The chemiluminescence was visualized by exposing to film (Kodak, Rochester, NY).

### 2.8. Statistical Analysis of Biological Assays

Each experiment was observed in triplicate. The data are presented as mean ± standard deviation (SD) of three independent experiments. Statistical differences were determined using Student's *t*-test and the STATA 11 software (version 11.1; StataCorp, College Station, TX, USA). All comparisons are made relative to untreated controls. A statistical difference was considered at ^*∗*^ *P* < 0.05.

## 3. Results

### 3.1. Effects of Peniocerol and Macdougallin on Viability of HCT-15 and MCF-7 Cells

Cytotoxic effect of peniocerol (**3**) and macdougallin (**4**) was tested against HCT-15 and MCF-7 cells using the crystal violet colorimetric method. Doxorubicin was used as a positive control. HCT-15 and MCF-7 cells were exposed to 10–80 *μ*M concentrations range of** 3** or** 4** for 24 or 48 h. Bioactivity of** 3** and** 4** was determined on the concentration that induced 50% inhibition on the growth of the treated cells as compared to the controls. The results are showed in Table 1.

### 3.2. Annexin V/Propidium Iodide Assay

**Figure 1:**
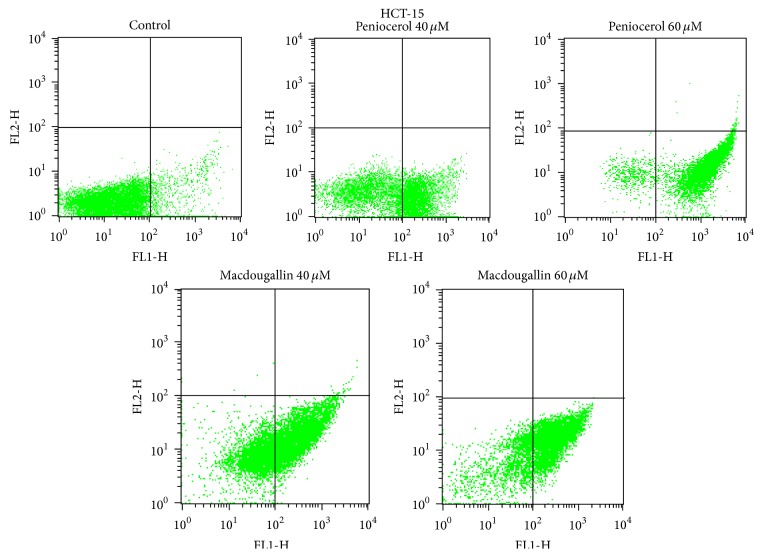
Annexin V/PI assay. HCT-15 cells treated with peniocerol (**3**) or macdougallin (**4**) at 40 and 60 *μ*M concentrations. Values are expressed as mean + S.D. of three independent experiments, each made in triplicate.

The HCT-15 cells were incubated with peniocerol (**3**) or macdougallin (**4**) at 40 and 60 *μ*M concentration during 24 h. As expected, apoptosis was sterol concentration dependent (Figure 1). The percentages of apoptosis induced by** 3** or** 4** in HCT-15 cell line are shown in Table 2.

**Figure 2:**
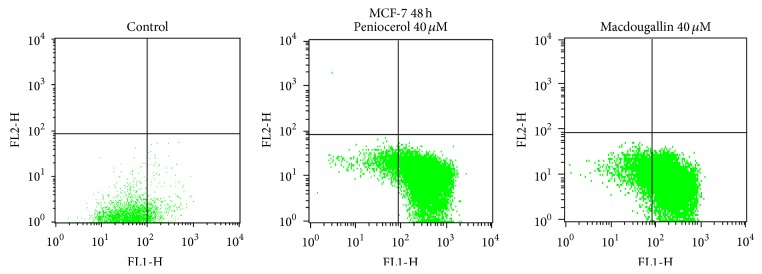
Annexin V/PI assay. MCF-7 cells treated with peniocerol and macdougallin at 48 h (40 *μ*M each one).

On the other hand, the MCF-7 cells were incubated with peniocerol (**3**) or macdougallin (**4**) at 40 *μ*M concentration during 48 h (Figure 2). The percentages of apoptosis induced by** 3** or** 4** in MCF-7 cell line are shown in Table 3.

### 3.3. Cell Cycle Analysis

**Figure 3:**
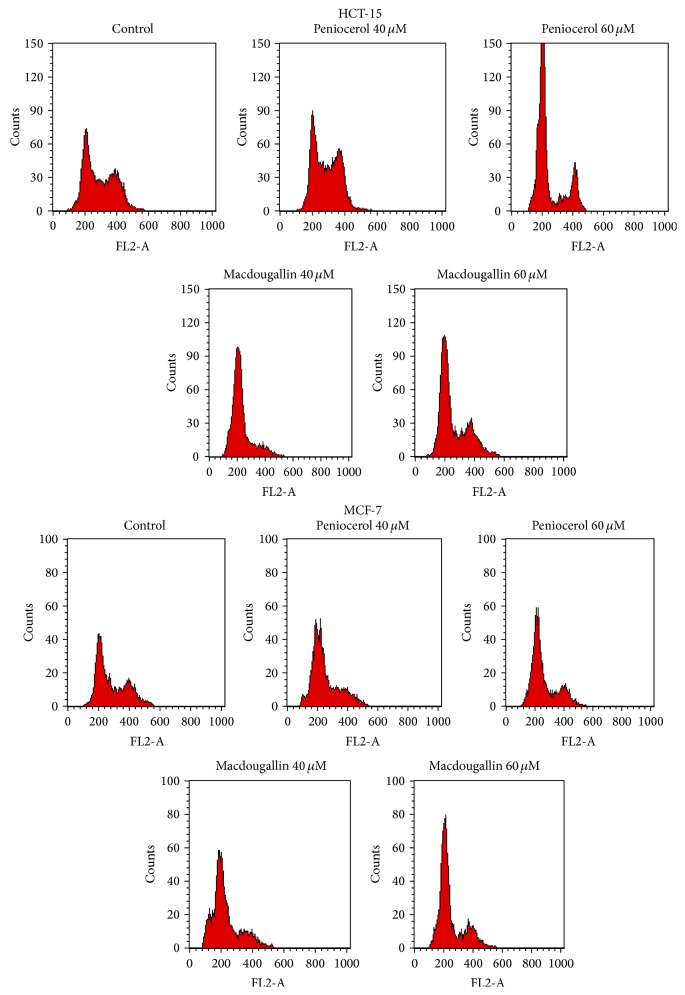
Effects of peniocerol and macdougallin on HCT-15 and MCF-7 cell cycle distribution.

The HCT-15 and MCF-7 cell cycle distribution changed compared with the control group when they were treated with peniocerol (**3**) or macdougallin (**4**) (Table 4). An increase of** 3** or** 4** concentration leads to an increase of the percentage of G_0_/G_1_ phase cells in both cell lines. For example, in HCT-15 cells at 40 *μ*M concentration of peniocerol the percentage of G_0_/G_1_ was 80.63 ± 0.61, while, at 60 *μ*M, it was 84.13 ± 2.30. A very similar effect was shown in the MCF-7 cell line (Table 4). Conversely, in HCT-15 cells, the S phase percentage was reduced as consequence of peniocerol treatment; at 40 *μ*M concentration, the percentage was 13.92 ± 0.85%, while, at 60 *μ*M concentration, it was 6.39 ± 4%. The same effect was seen in MCF-7. In general,** 4** showed the same behavior in both cell lines. These results showed that** 3** and** 4** induced HCT-15 and MCF-7 cell cycle arrest that occurs mainly in G_0_/G_1_ phase (Figure 3).

### 3.4. Peniocerol (**3**) and Macdougallin (**4**) Induced Cleavage of PARP in HCT-15 and MCF-7 Cells

**Figure 4:**
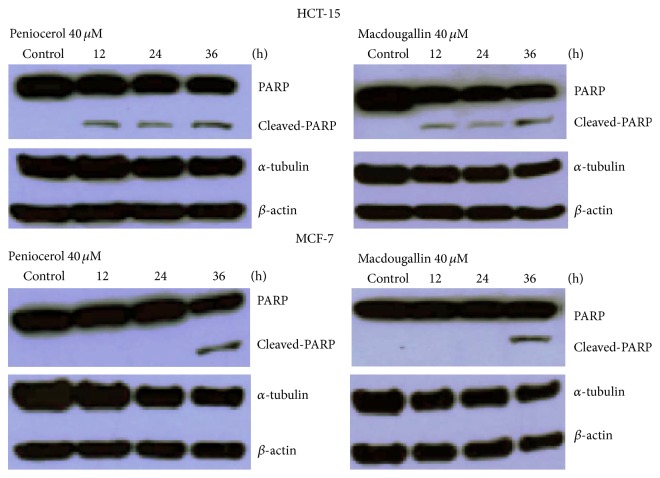
PARP cleaved followed by treatment with peniocerol and macdougallin.

To further validate apoptotic effect of** 3** and** 4** on the HCT-15 and MCF-7 cells, we examined the PARP cleavage by Western blotting assay after treatment at 12, 24, and 36 h. The concentrations used were 40 *μ*M for both sterols. The results showed that, in the HCT-15 cells, the PARP cleavage is time-dependent (Figure 4). These results suggest that peniocerol and macdougallin have similar ability to induce PARP cleavage.

## 4. Discussion

Both peniocerol and macdougallin have been shown to inhibit the proliferation of U-251, PC-3, K-562, HCT-15, and MCF-7 human cancer lines [13]. Using HCT-15 and MCF-7 cells in this paper we showed that cell death induced by** 3** and** 4** is by apoptosis.

Apoptosis plays an important role in the maintenance of tissue homeostasis by the selective elimination of excess cells. Furthermore, apoptosis induction of cancer cells is also recognized to be useful in cancer treatment, since some anticancer drugs such as etoposide, cisplatin, and paclitaxel are known to induce apoptosis in target cells [15].

**Figure 5:**
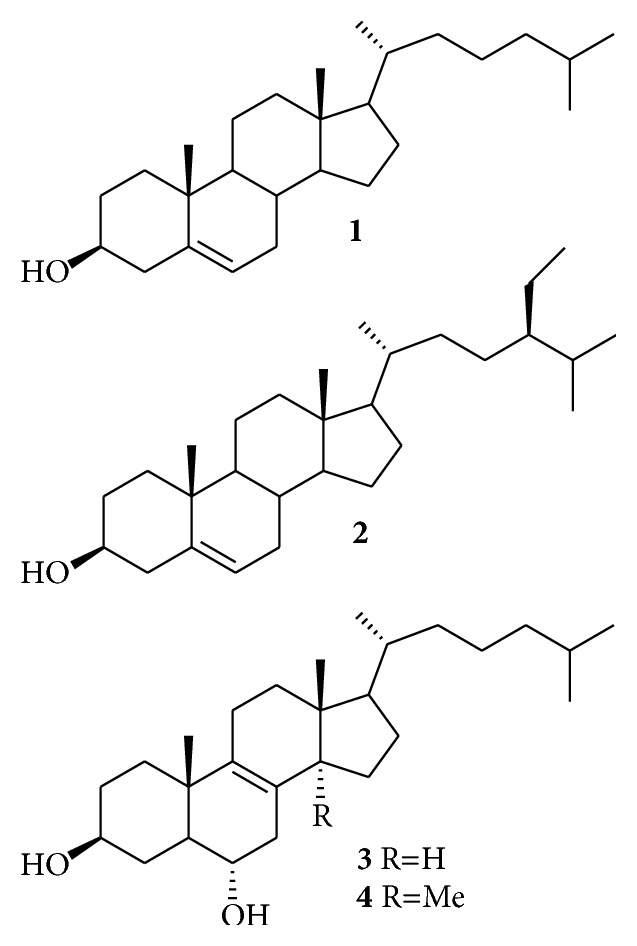


In recent years, several studies performed* in vitro* have shown that the cytotoxic effects of several sterols are mediated by apoptosis [16, 17]. In agreement with these observations, the Annexin V/Propidium Iodide double-staining results presented here showed that cell death induced by** 3** and** 4** (Figure 5) is also due to apoptosis (Tables 2 and 3).

On the other hand, it is known that PARP is a substrate for certain caspases that get activated during early stages of apoptosis, and then the detection of cleavage of PARP serves as a marker of apoptosis. Our results showed that** 3** and** 4** induced cleavage of PARP in HCT-15 and MCF-7 cells (Figure 4).

Additionally, both peniocerol (**3**) and macdougallin (**4**) induced HCT-15 and MCF-7 cell cycle arrest that occurs in G_0_/G_1_ phase (Figure 2). This result suggests the possibility that** 3** and** 4** could be useful for the control of cancer growth [18].

It is worth noting that the only structural difference between peniocerol and macdougallin is the presence of *α*-methyl group at C-14 in the latter. Since both sterols showed the same biological activities, then the presence of the C-14 methyl group in the macdougallin structure is not relevant for its biological activities.

Phytosterols have been shown to promote apoptosis, an important mechanism in the inhibition of carcinogenesis. An increased apoptosis of prostate cancer cells has been observed by 73% on *β*-sitosterol treatment at a dosage of 16 *μ*M. Likewise, it has been observed that treatment of human leukemia cells with *β*-sitosterol at varying concentrations for 72 h increased the percentage of apoptotic cells in a dose-dependent manner.

The mechanism by which *β*-sitosterol promotes apoptosis has been investigated and it has been shown that treatment of human leukemia cells with *β*-sitosterol resulted in an increased activity of caspase-3 in a dose-dependent manner [16]. However, it has been reported that the intake of *β*-sitosterol could accelerate intestinal tumorigenesis in Apc^Min^ which makes the intake of *β*-sitosterol somewhat hazardous [12].

Although we have not shown the exact mechanism of apoptosis induced by macdougallin and peniocerol, there is a possibility that** 3** and** 4** could induce programmed cell death in a similar way as *β*-sitosterol does.

*M. geometrizans*, known as “garambullo,” is a medicinal plant used by the Otomi and Mixtec ethnic groups as anti-inflammatory remedy; our previous studies showed that sterols** 3** and** 4** are potent anti-inflammatory agents; these findings supported the traditional use of this species [13]. It is known that inflammatory cells and inflammatory mediators are present in almost all tumor types irrespective of the trigger for development [19]. Then it has been proposed that anti-inflammatory agents could help treat cancer; for example, it has been reported that daily use of aspirin is associated with a significant reduction in the incidence of colorectal adenomas in patients with previous colorectal cancer [20].

## 5. Conclusions

If we take into account both anti-inflammatory properties and their apoptotic effects, it is feasible to assume that sterols** 3** and** 4** might be considered as prototypes for possible development of new anticancer agents.

## Supplementary Material

The ^1^H and ^13^C nmr spectra as well as HPLC analyses of peniocerol and macdougallin are showed in the Supplementary Material, also the RX analyses of peniocerol is showed.

## Figures and Tables

**Table 1:** Cytotoxicity (IC_50_) of peniocerol and macdougallin in HCT-15 and MCF-7 cancer cells.

Compound	Human cancer cell lines	IC_50_ value (*μ*M)	Incubation time (hour)
Peniocerol	HCT-15	41.66 ± 0.41	24
		28.85 ± 0.60	48
	MCF-7	48.17 ± 0.35	24
		21.77 ± 0.39	48

Macdougallin	HCT-15	37.67 ± 0.76	24
		33.71 ± 0.84	48
	MCF-7	31.83 ± 1.06	24
		28.15 ± 0.54	48

Doxorubicin (DOX)	HCT-15	0.36 ± 0.03	24
		0.16 ± 0.02	48
	MCF-7	0.37 ± 0.02	24
		0.28 ± 0.01	48

^*∗*^HCT-15: colorectal adenocarcinoma. MCF-7: breast adenocarcinoma.

^*∗*^Values are expressed as mean ± S.D of three independent experiments, each made in triplicate.

**Table 2:** Percentage of apoptosis induced by peniocerol or macdougallin in HCT-15 cell line.

Treatment	Percentage of apoptosis (%)
(A) Control, HCT-15, 24 h	7.14 ± 1.13% (EA)
	0.24 ± 0.11% (LA)

(B) Peniocerol 40 *μ*M, 24 h	51.96 ± 5.54% (EA)
	0.11 ± 0.06% (LA)

(C) Peniocerol 60 *μ*M, 24 h	73.36 ± 4.20% (EA)
	14.60 ± 2.53% (LA)

(D) Macdougallin 40 *μ*M, 24 h	63.30 ± 0.42% (EA)
	1.39 ± 0.22% (LA)

(E) Macdougallin 60 *μ*M, 24 h	77.63 ± 2.81% (EA)
	0.04 ± 0.05% (LA)

EA: early apoptosis, LA: late apoptosis.

Values are expressed as mean + S.D. of three independent experiments, each made in triplicate.

**Table 3:** Percentage of apoptosis induced by peniocerol or macdougallin in MCF-7 cell line.

Treatment	Percentage of apoptosis (%)
(A) Control, MCF-7, 48 h	11 ± 3% (EA)
	0.36 ± 0.15 (LA)

(B) Peniocerol 40 *μ*M, 48 h	83 ± 2% (EA)
	0.23 ± 0.12 (LA)

(C) Macdougallin 40 *μ*M, 48 h	66 ± 3% (EA)
	1.27 ± 6% (LA)

EA: early apoptosis, LA: late apoptosis.

Values are expressed as mean ± S.D. of three independent experiments, each performed in triplicate (*n* = 9).

**Table 4:** Effects of peniocerol and macdougallin on cell cycle distribution.

Compound	Cell cycle phase (%)					
	HCT-15			MCF-7		
	G1	S	G2/M	G1	S	G2/M
Control (DMSO)						
24 h	49.82 ± 0.61	22.05 ± 0.14	28.72 ± 0.40	59.6 ± 0.78	15.61 ± 0.86	25.22 ± 0.36

Peniocerol (1)						
40 *μ*M	80.63 ± 0.61^*∗*^	13.92 ± 0.85^*∗*^	5.56 ± 0.22^*∗*^	75.29 ± 1.65^*∗*^	8.93 ± 1.05^*∗*^	16.10 ± 0.80^*∗*^
60 *μ*M	84.13 ± 2.30^*∗*a^	6.39 ± 4.21^*∗*a^	5.84 ± 3.49^*∗*^	78.49 ± 0.70^*∗*a^	6.53 ± 0.08^*∗*a^	15.19 ± 0.72^*∗*^

Macdougallin (2)						
40 *μ*M	64.17 ± 5.39^*∗*^	10.89 ± 0.79^*∗*^	25.22 ± 4.60^*∗*^	78.34 ± 1.28^*∗*^	12.49 ± 1.19^*∗*^	9.55 ± 1.49^*∗*^
60 *μ*M	68.61 ± 2.65^*∗*^	7.93 ± 0.12^*∗*a^	23.16 ± 2.62^*∗*^	80.28 ± 1.04^*∗*^	10.49 ± 0.59^*∗*a^	9.44 ± 0.57^*∗*^

Timidine						
2 mM	82.18 ± 1.09	13.84 ± 1.76	4.45 ± 1.94	70.16 ± 1.50	17.34 ± 0.67	13.05 ± 1.24

Nocodazol						
100 ng/mL	9.2 ± 0.85	17.59 ± 1.59	73.63 ± 2.51	3.90 ± 0.92	3.95 ± 1.80	92.21 ± 1.54

Values are expressed as mean + S.D. of three independent experiments, each made in triplicate. Untreated cells were used as a control. ^*∗*^Significant difference with untreated group (*P* < 0.05). ^a^Significant difference with treated group at 24 h (*P* < 0.05).
