# Supplementary material for: Effect of Sterols Isolated from Myrtillocactus geometrizans on Growth Inhibition of Colon and Breast Cancer Cells
Source: Evid Based Complement Alternat Med. 2015 May 31;2015:589350. doi: 10.1155/2015/589350 (PMC4465765; doi:10.1155/2015/589350)
Supplement: Supplementary file 1 — The 1H and 13C nmr spectra as well as HPLC analyses of peniocerol and macdougallin are showed in the Supplementary Material, also the RX analyses of peniocerol is showed. [file 589350.f1.pdf]

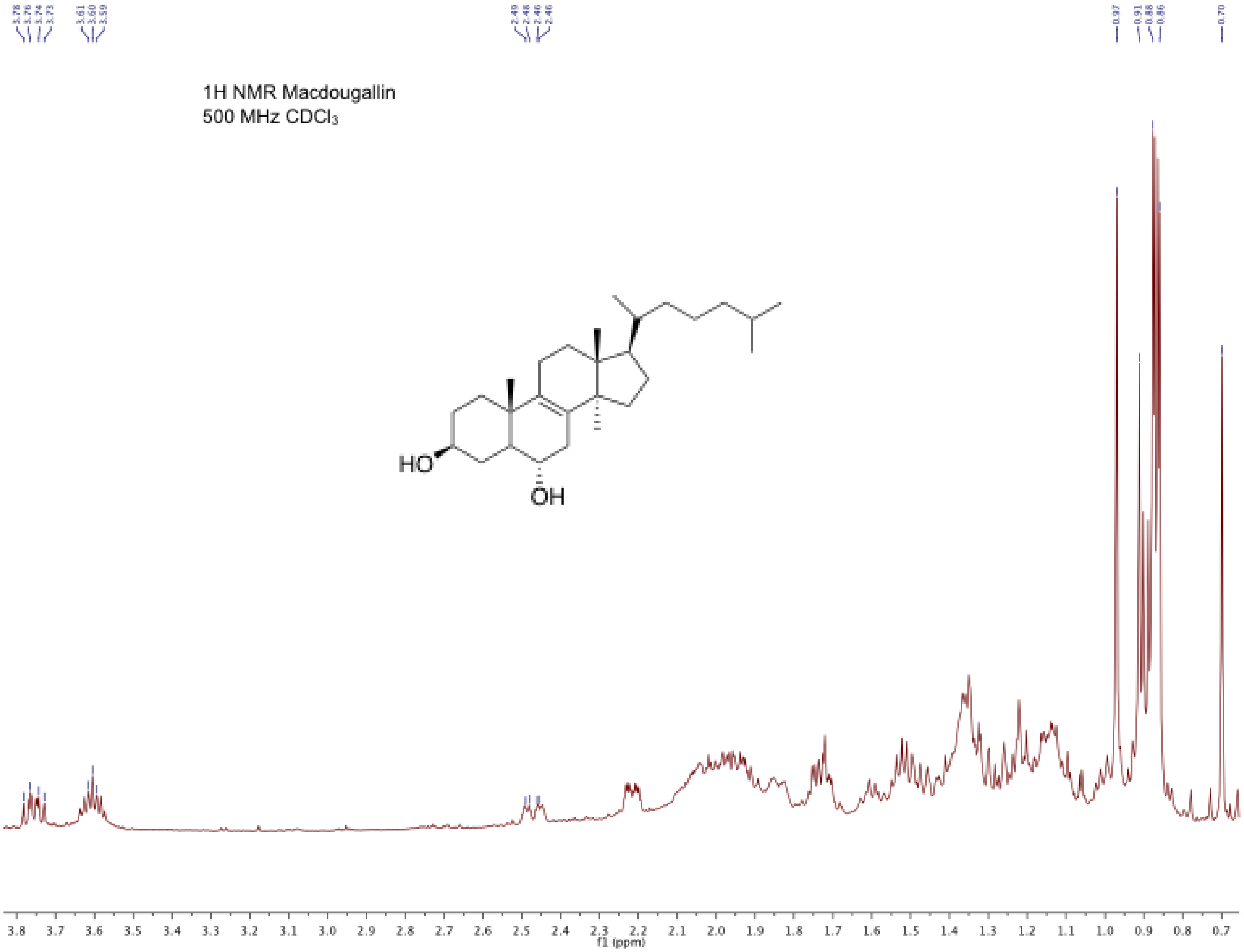
1H NMR Macdougallin
500 MHz $CDCl_3$
HO
OH
f1 (ppm)

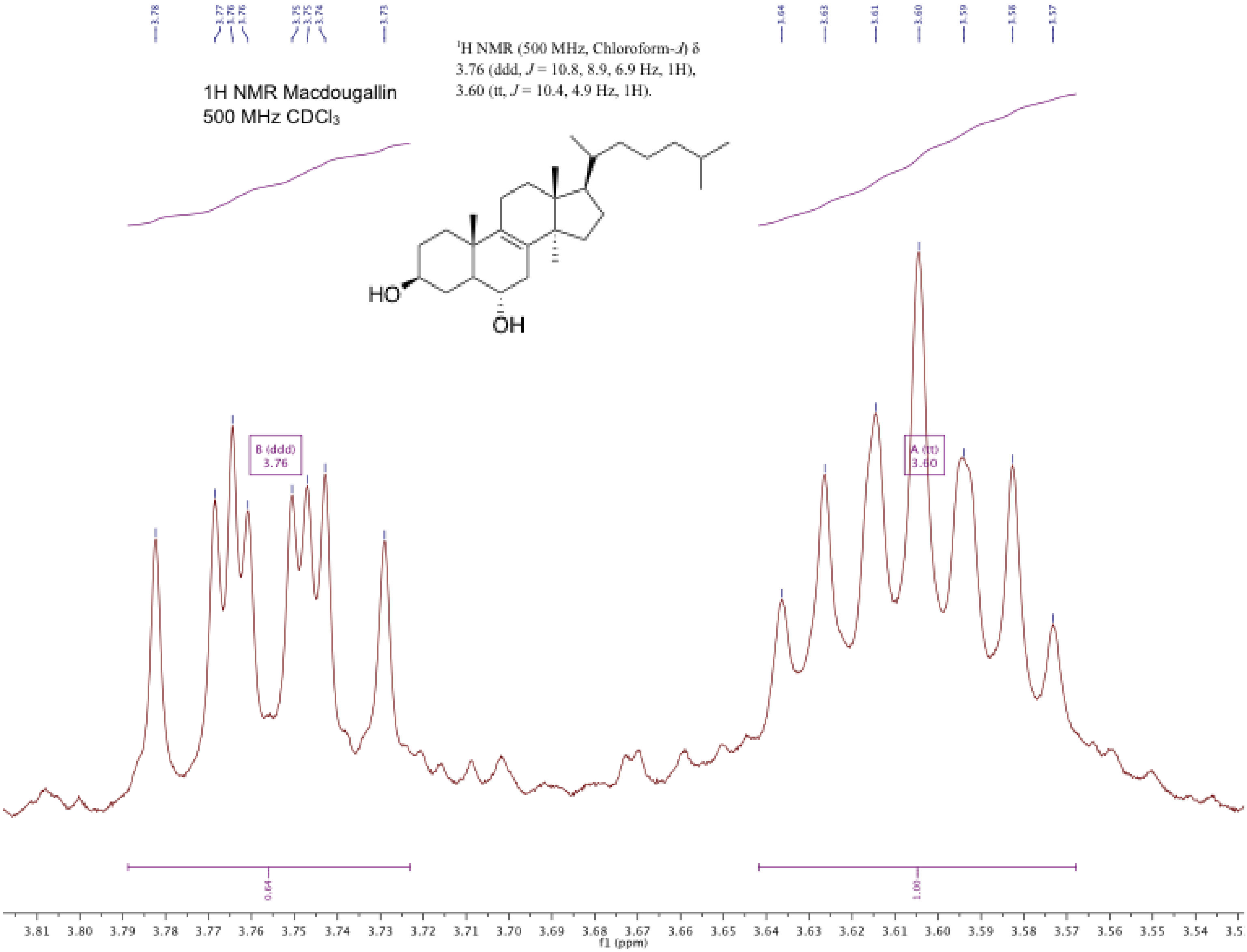

1H NMR Macdougallin
500 MHz CDCl3
1H NMR (500 MHz, Chloroform-d) δ
3.76 (ddd, J = 10.8, 8.9, 6.9 Hz, 1H),
3.60 (tt, J = 10.4, 4.9 Hz, 1H).
HO
OH
B (ddd)
3.76
A (tt)
3.60
0.64
1.00
f1 (ppm)

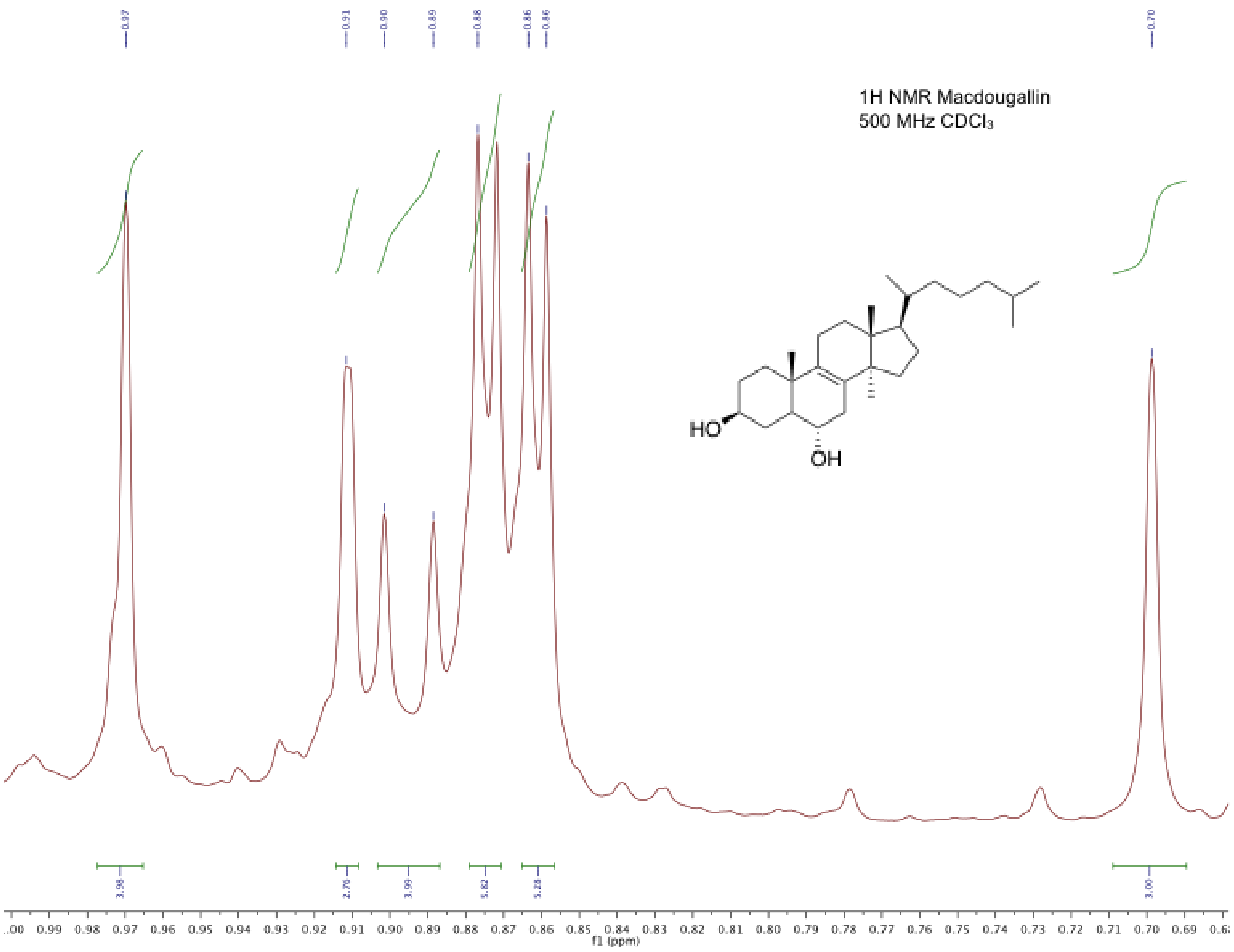

1H NMR Macdougallin
500 MHz CDCl3
HO
OH
0.97
0.91
0.90
0.89
0.88
0.86
0.86
0.70
3.98
2.76
3.99
5.82
5.28
3.00
f1 (ppm)

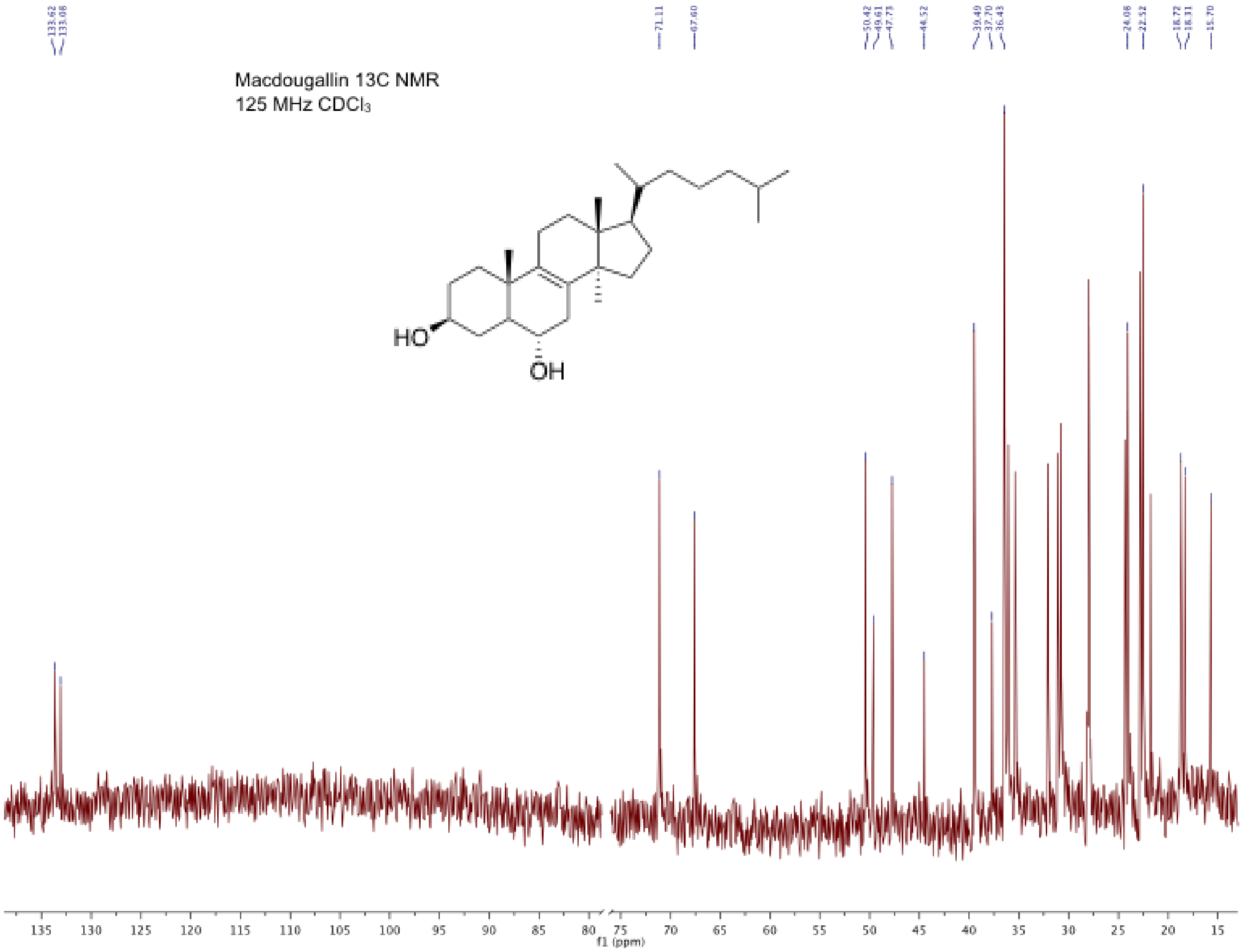
Macdougallin 13C NMR
125 MHz $CDCl_3$
133.62
133.08
71.11
67.60
50.42
49.61
47.73
44.52
39.49
37.70
36.43
24.08
22.52
18.72
18.31
15.70
HO
OH
f1 (ppm)

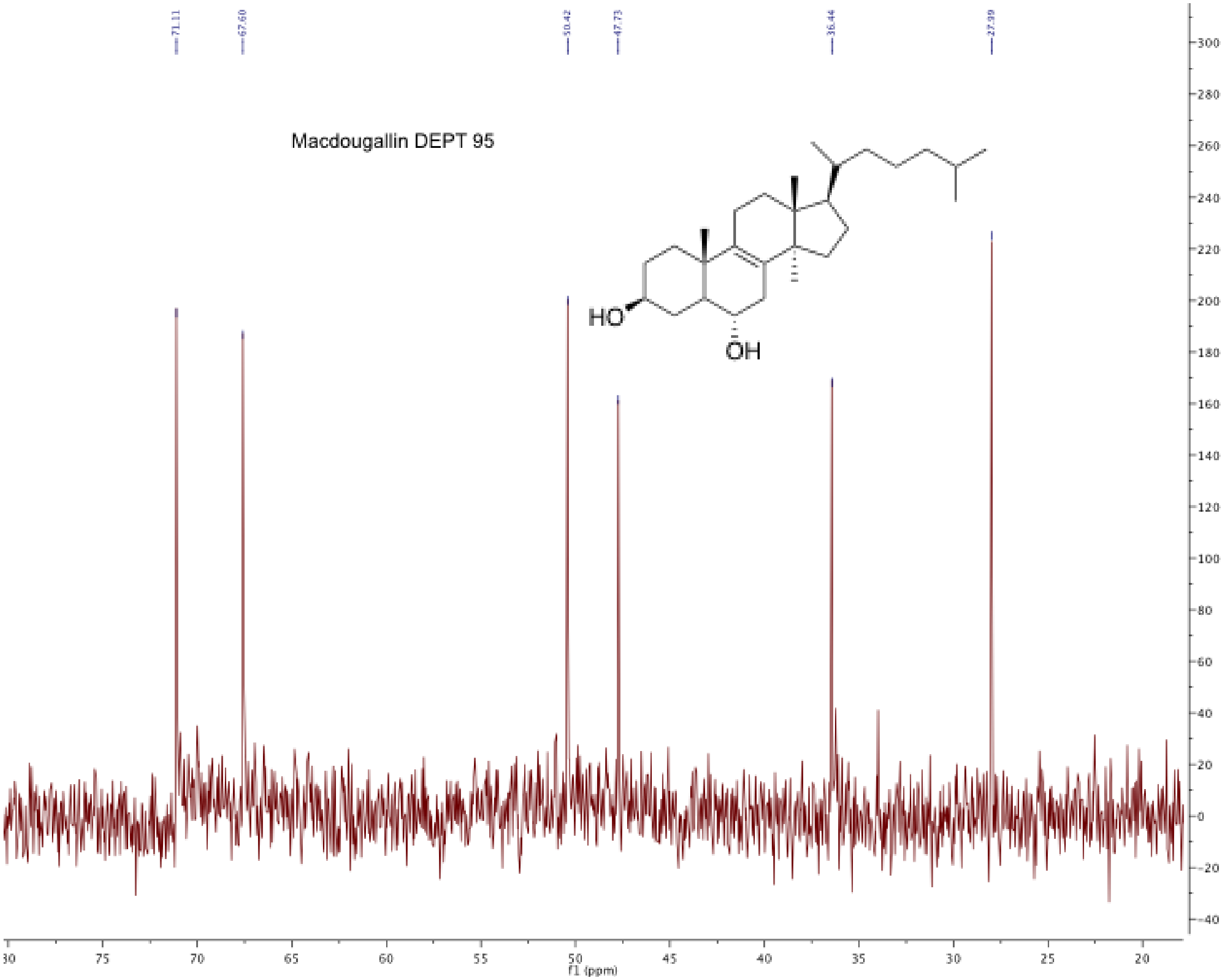

Macdougallin DEPT 95
71.11
67.60
50.42
47.73
36.44
27.99
HO
OH
f1 (ppm)

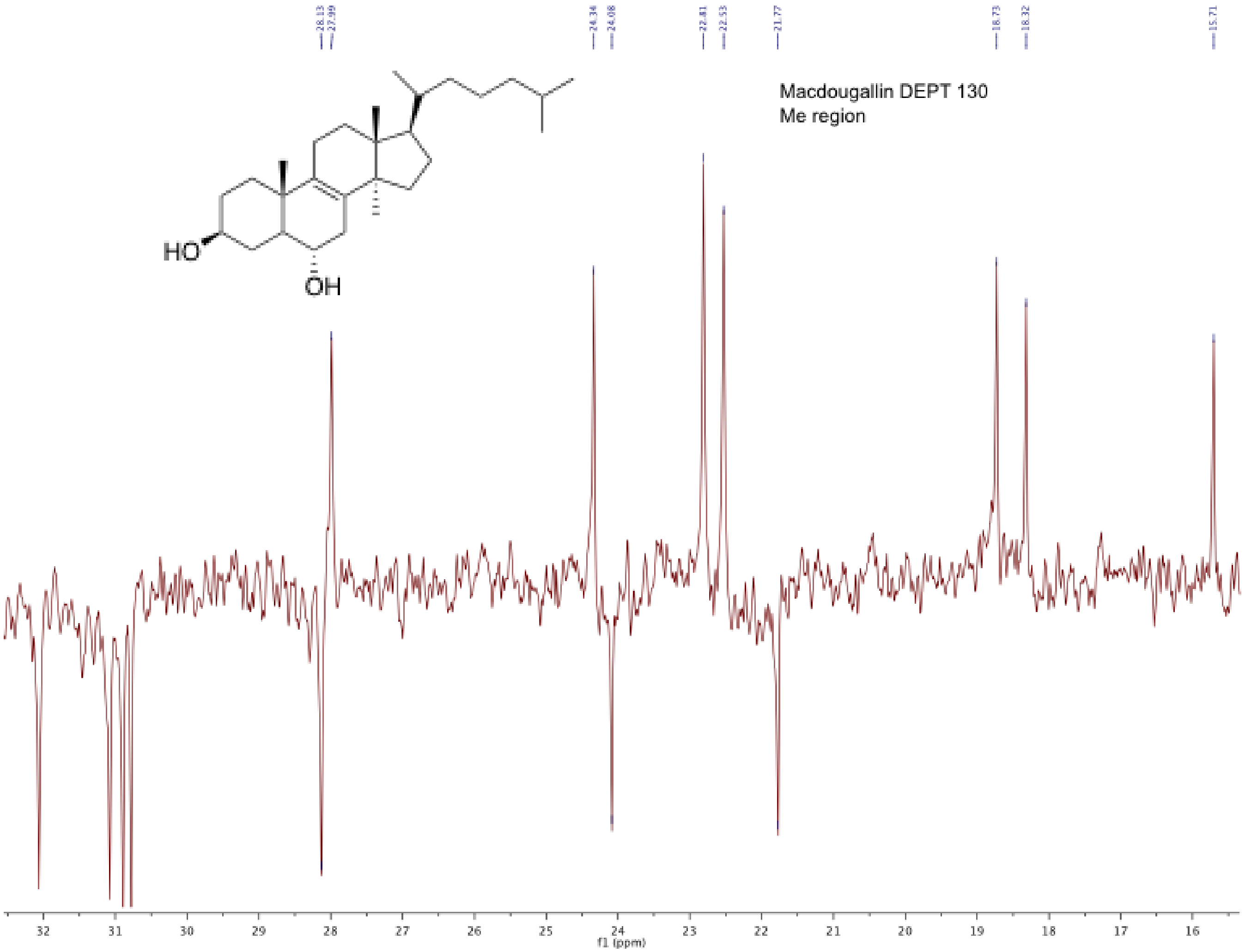

Macdougallin DEPT 130
Me region
HO
OH
28.13
27.99
24.34
24.08
22.81
22.53
21.77
18.73
18.32
15.71
32
31
30
29
28
27
26
25
24
23
22
21
20
19
18
17
16
f1 (ppm)

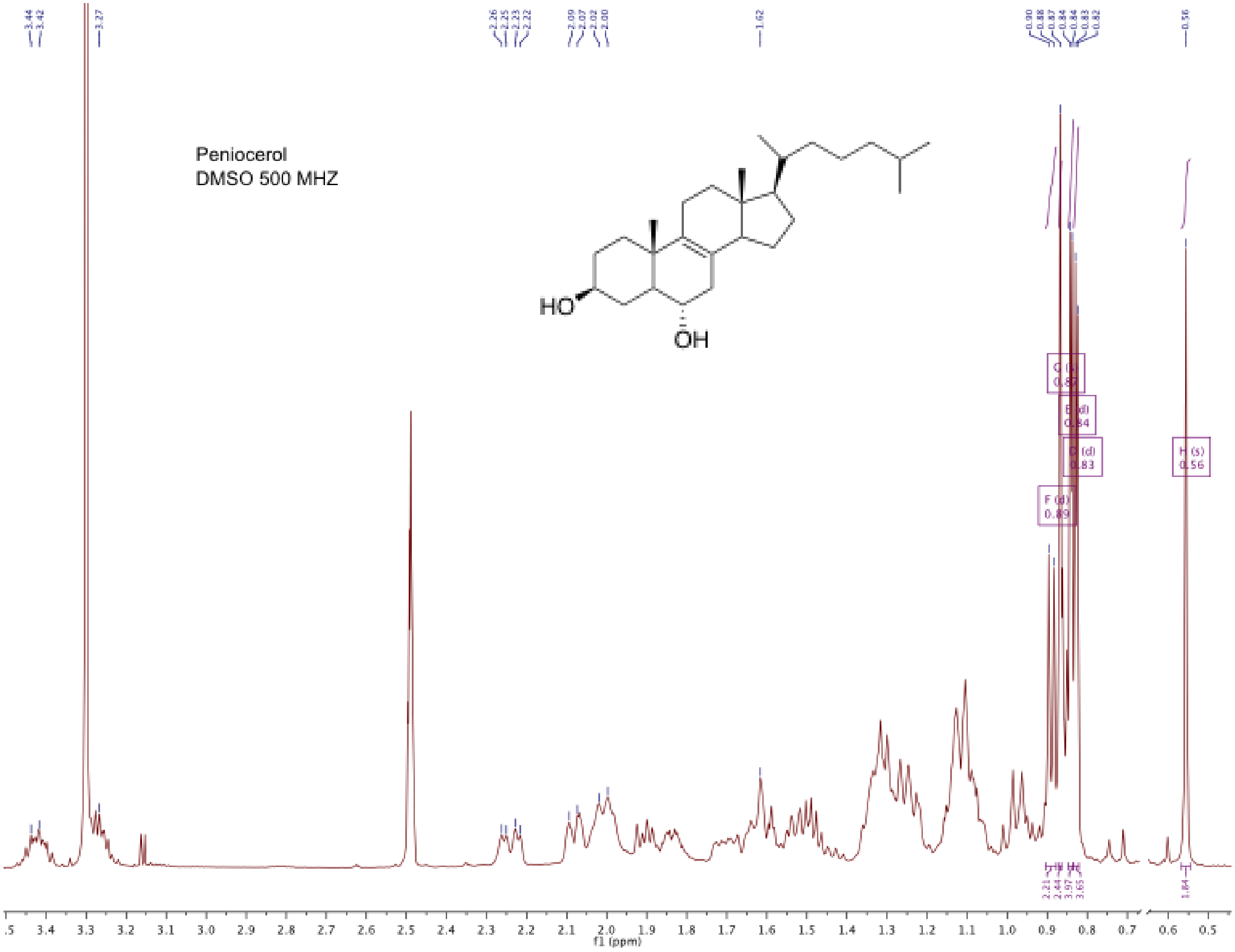

Peniocerol
DMSO 500 MHZ
HO
OH
f1 (ppm)

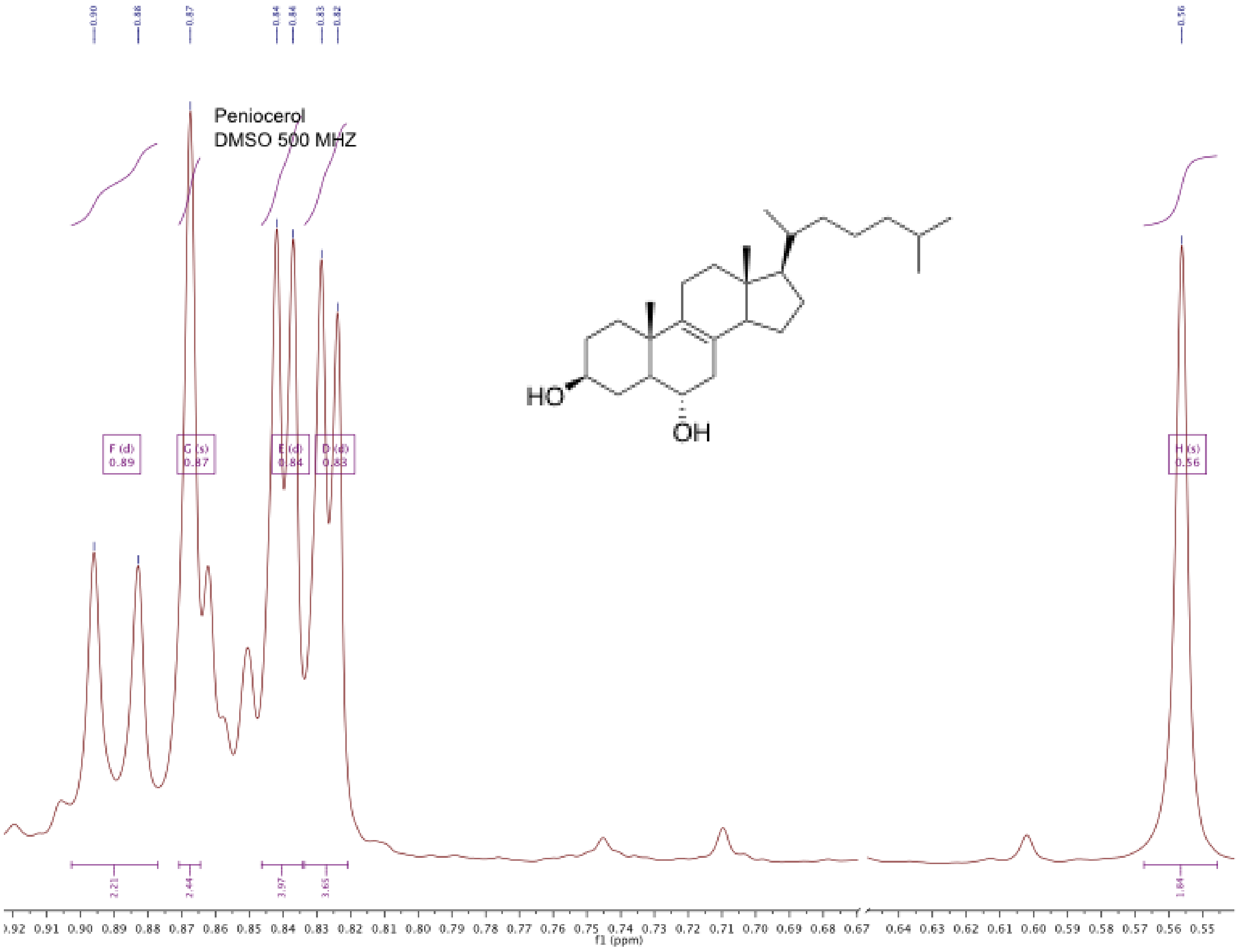

Peniocerol
DMSO 500 MHZ
HO
OH
F (d) 0.89
G (s) 0.87
E (d) 0.84
D (d) 0.83
H (s) 0.56
2.21
2.44
3.97
3.65
1.84
f1 (ppm)

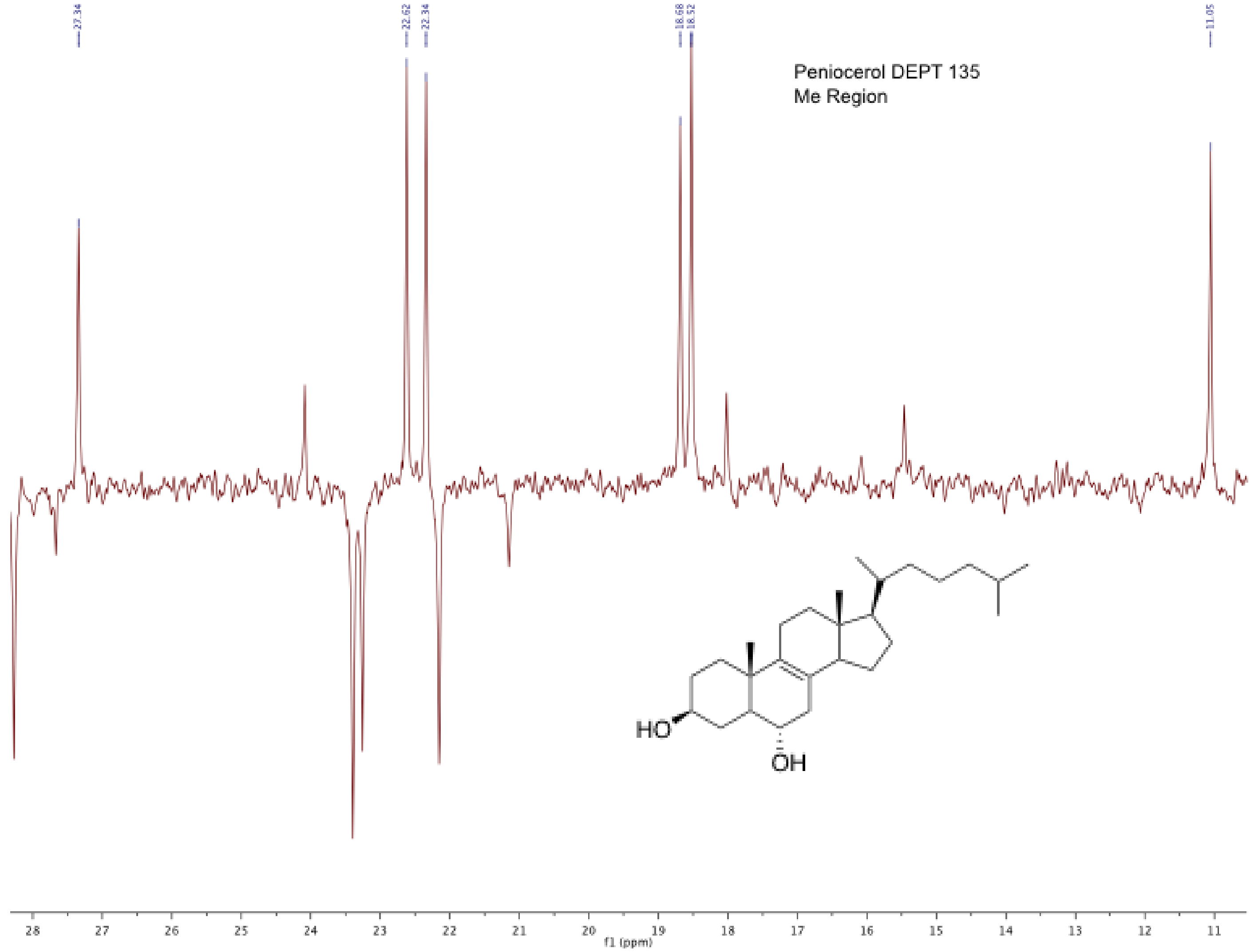
27.34
22.62
22.34
18.68
18.52
11.05
Peniocerol DEPT 135
Me Region
HO
OH
28
27
26
25
24
23
22
21
20
19
18
17
16
15
14
13
12
11
f1 (ppm)

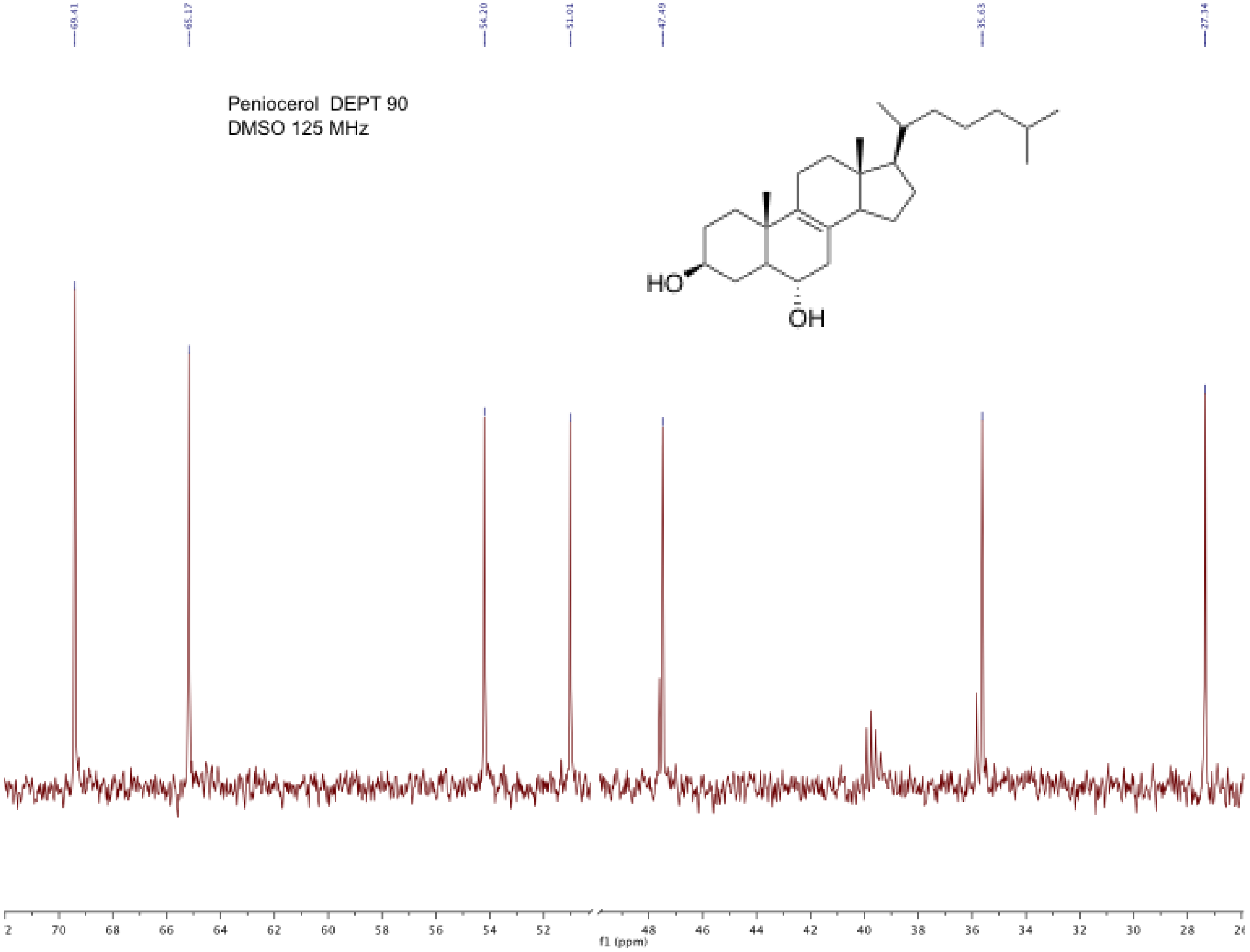

69.41
65.17
54.20
51.01
47.49
35.63
27.34
Peniocerol DEPT 90
DMSO 125 MHz
HO
OH
f1 (ppm)

Peniocerol´s structure determinate by RX

Table 1. Crystal data and structure refinement for **Mgmr**.

Sample code	252MVM08	
Project Title	**Mgmr**	
Empirical formula	$C_{27} H_{46} O_2 CH_3OH$	
Formula weight	434.68	
Temperature	298(2) K	
Wavelength	0.71073 Å	
Crystal system	Orthorhombic	
Space group	$P\ 2_1\ 2_1\ 2_1$	
Unit cell dimensions	a = 8.147(3) Å	α= 90°.
	b = 9.137(3) Å	β= 90°.
	c = 36.585(14) Å	γ = 90°.
Volume	2723.3(18) $Å^3$	
Z	4	
Density (calculated)	1.060 Mg/m^3	
Absorption coefficient	0.066 mm^{-1}	
F(000)	968	
Crystal size / shape / color	0.30 x 0.07 x 0.05 mm / Prism/ Colorless	
Theta range for data collection	2.23 to 25.68°.	
Diffractometer used /Scan Mode	Bruker Smart APEX AXS CCD area detector/ omega scans	
Index ranges	-9<=h<=9, -11<=k<=10, -44<=l<=44	
Reflections collected	29873	
Independent reflections	5129 [R(int) = 0.1862]	
Completeness to theta = 25.68°	99.7 %	
Absorption correction	None	
Refinement method	Full-matrix least-squares on F^2	
Data / restraints / parameters	5129 / 3 / 295	
Goodness-of-fit on F^2	1.000	
Final R indices [I>2sigma(I)]	R1 = 0.1031, wR2 = 0.1219	
R indices (all data)	R1 = 0.2490, wR2 = 0.1637	
Absolute structure parameter	Not determined.	
Largest diff. peak and hole	0.158 and -0.129 e $Å^{-3}$	
Solved by	Simon Hernandez-Ortega	

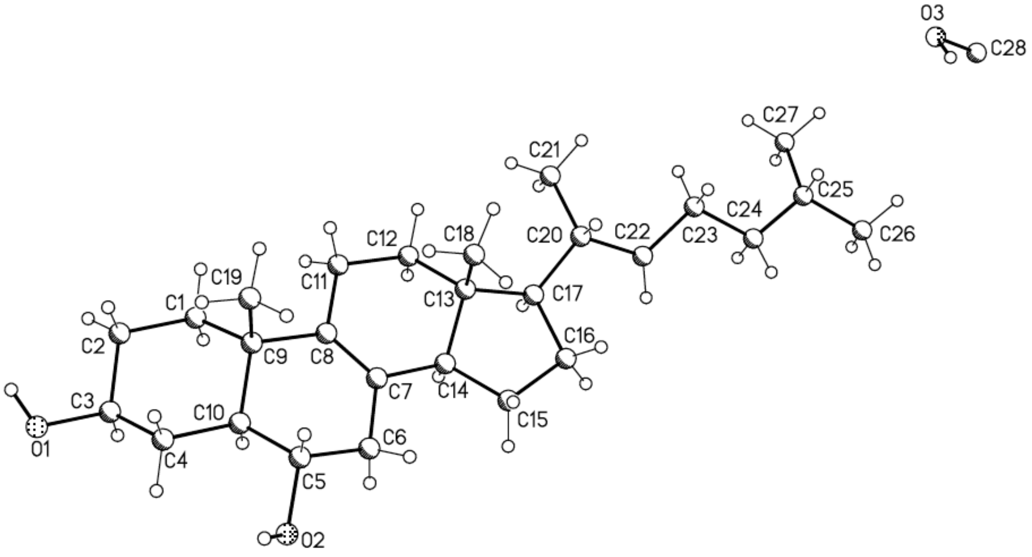
O3
C28
C27
C21
C25
C20
C22
C23
C24
C26
C12
C18
C11
C13
C17
C1
C19
C16
C2
C9
C8
C7
C14
C3
C10
C6
C15
O1
C4
C5
O2

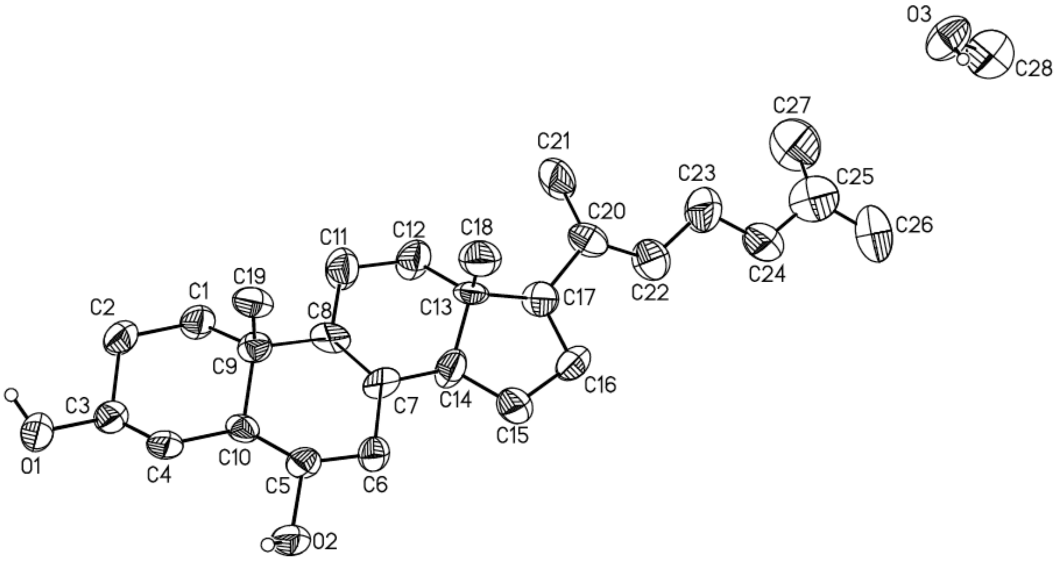
O3
C28
C27
C21
C23
C25
C20
C26
C11
C12
C18
C24
C22
C1
C19
C13
C17
C2
C8
C9
C16
C7
C14
C3
C15
O1
C4
C10
C5
C6
O2

Data File C:\CHEM32\1\DATA\MVM00009.D
Sample Name: MCMACDO

===

Acq. Operator   : Erendira Garcia
Acq. Instrument : CL6-LCR-IQUI   Location : Vial 1
Injection Date : 3/20/2015 4:56:44 PM
Acq. Method   : C:\HPCHEM\1\METHODS\MARIANO.M
Last changed   : 3/20/2015 3:25:27 PM by Erendira Garcia
                  (modified after loading)
Analysis Method : C:\CHEM32\1\METHODS\123.M
Last changed   : 3/20/2015 4:15:38 PM by CARMEN
                  (modified after loading)
Sample Info   : Eclipse Plus C18 3.5um 100x2.1mm flujo 0.2 mL/min 254 nm
                  Agua/ACN Inicial 80/20 20 min 0/100

DAD1 C, Sig=254,16 Ref=360,100 (MVM00009.D)

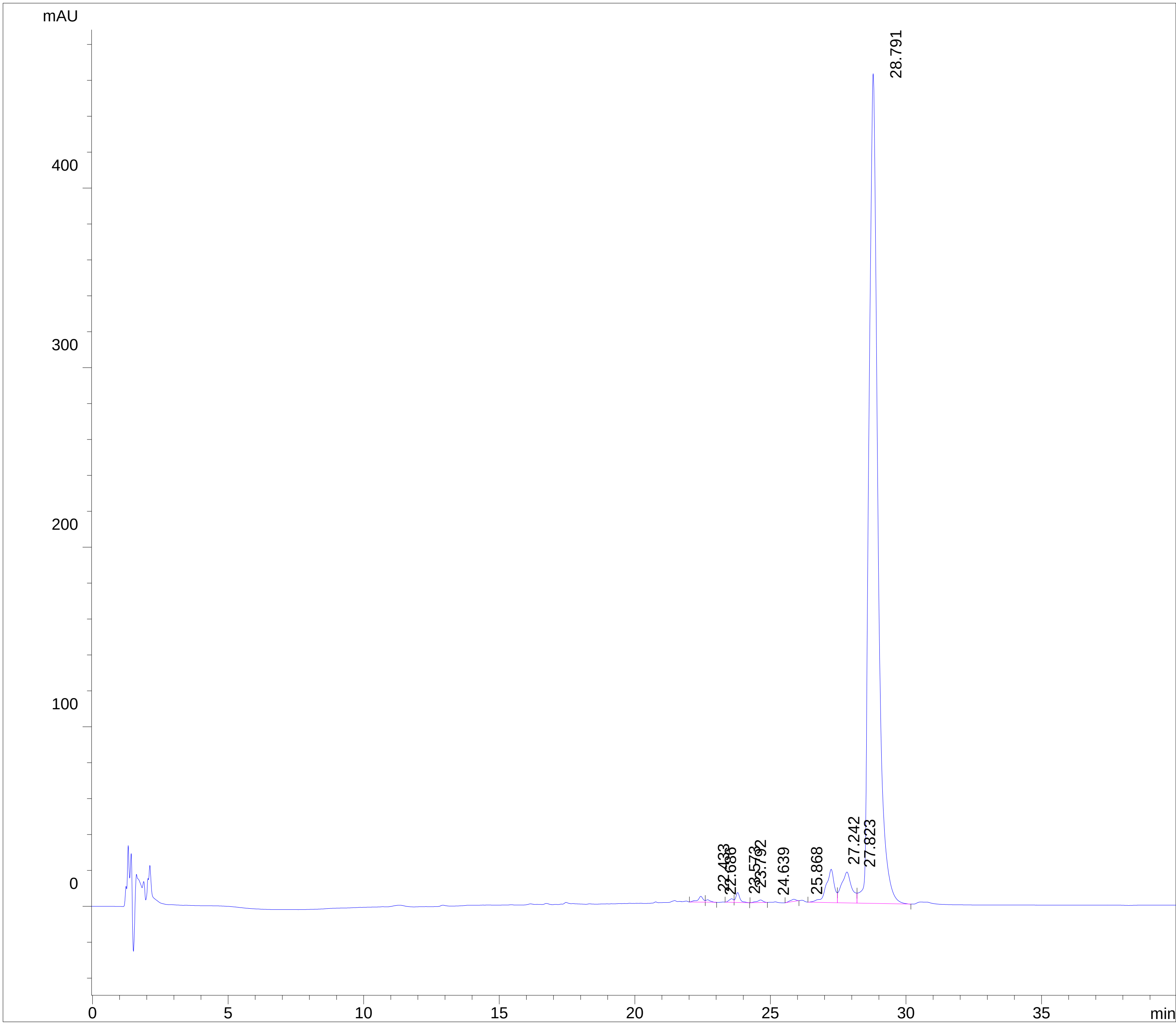

===

Area Percent Report

===

Sorted By : Signal
Multiplier: : 1.0000
Dilution: : 1.0000
Use Multiplier & Dilution Factor with ISTDs

Signal 1: DAD1 C, Sig=254,16 Ref=360,100

Peak #	RetTime [min]	Type	Width [min]	Area [mAU*s]	Height [mAU]	Area %
1	22.433	BV	0.1919	43.96069	3.24812	0.3798
2	22.686	VB	0.1573	15.55498	1.38220	0.1344
3	23.573	BV	0.1635	21.66733	1.98282	0.1872
4	23.792	VB	0.1538	57.87674	5.54340	0.5001
5	24.639	BB	0.2283	22.81826	1.44417	0.1972
6	25.868	BB	0.2154	18.23200	1.19906	0.1575
7	27.242	BV	0.2839	388.60580	18.58577	3.3577
8	27.823	VV	0.3577	453.43222	17.19118	3.9178
9	28.791	VB	0.3224	1.05515e4	462.12866	91.1683

Totals : 1.15736e4 512.70538

===

*** End of Report ***

Data File C:\CHEM32\1\DATA\MVM00008.D
Sample Name: Penf2

===

Acq. Operator   : Erendira Garcia
Acq. Instrument : CL6-LCR-IQUI   Location : Vial 1
Injection Date : 3/20/2015 3:41:44 PM
Acq. Method   : C:\HPCHEM\1\METHODS\MARIANO.M
Last changed   : 3/20/2015 3:25:27 PM by Erendira Garcia
                  (modified after loading)
Analysis Method : C:\CHEM32\1\METHODS\123.M
Last changed   : 3/20/2015 3:26:08 PM by CARMEN
Sample Info   : Eclipse Plus C18 3.5um 100x2.1mm flujo 0.2 mL/min 254 nm
                  Agua/ACN Inicial 80/20 20 min 0/100

DAD1 C, Sig=254,16 Ref=360,100 (MVM00008.D)

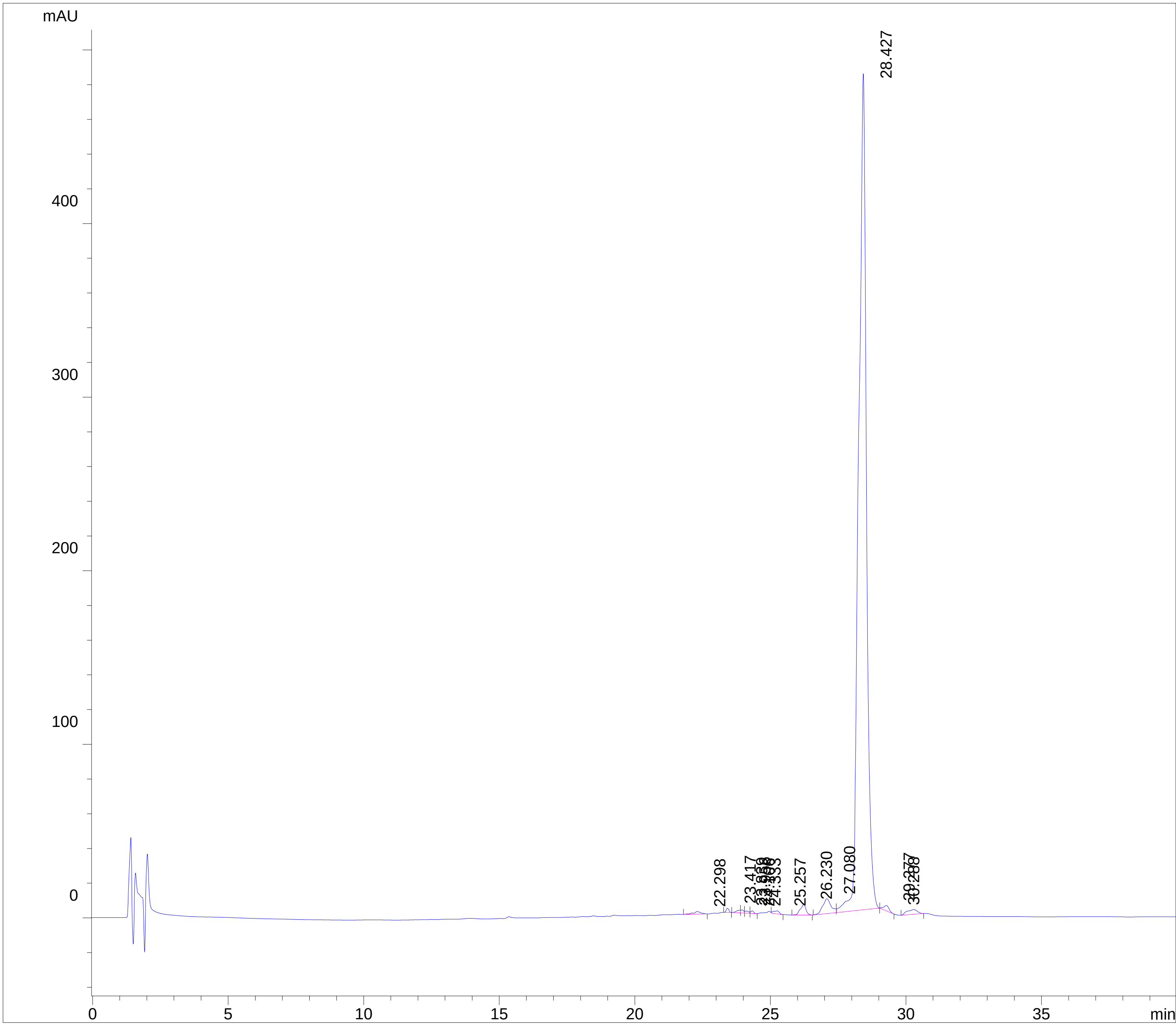

===

Area Percent Report

===

Sorted By : Signal
Multiplier: : 1.0000
Dilution: : 1.0000
Use Multiplier & Dilution Factor with ISTDs

Signal 1: DAD1 C, Sig=254,16 Ref=360,100

Peak #	RetTime [min]	Type	Width [min]	Area [mAU*s]	Height [mAU]	Area %
1	22.298	BB	0.2518	28.87656	1.50566	0.2849
2	23.417	BB	0.1036	16.44096	2.47249	0.1622
3	23.839	BV	0.1359	15.26783	1.53679	0.1506
4	23.956	VV	0.1055	13.32459	1.81870	0.1314
5	24.106	VV	0.1361	12.20759	1.22673	0.1204
6	24.333	VB	0.1044	10.71939	1.51783	0.1057
7	25.257	VB	0.1987	26.18956	1.78988	0.2584
8	26.230	BB	0.1944	88.25874	6.26259	0.8707
9	27.080	BV	0.3049	182.31735	8.54637	1.7985
10	28.427	VB	0.2772	9627.62500	481.68204	94.9748
11	29.277	BB	0.2056	42.91954	3.09767	0.4234
12	30.288	BB	0.3763	72.88666	2.70575	0.7190

Totals : 1.01370e4 514.16252

===

*** End of Report ***
